# How and why do young soccer players change the Flow State?

**DOI:** 10.1371/journal.pone.0233002

**Published:** 2020-05-14

**Authors:** Alfonso Castillo-Rodríguez, Christian Ureña Lopera, Wanesa Onetti-Onetti, José Luis Chinchilla-Minguet

**Affiliations:** 1 Department of Physical Education and Sports, University of Granada, Granada, Spain; 2 UNIR, Universidad Internacional de la Rioja, Logroño, España; 3 Department of the Languages, Arts and Sport Didactic, Universidad de Malaga, Andalucía-Tech, IBIMA, Malaga, Spain; Berner Fachhochschule, SWITZERLAND

## Abstract

Flow State (FS) as well as other psychological characteristics influence sports performance (SP) and could be relevant according to the playing position in team sports, such as the soccer where players have different specific functions within the team. The aim of this study was to evaluate the difference in FS dimensions in young soccer players between training time (TR) and official competition time (CM), according to the playing position and, to find relationships between FS dimensions and physical characteristics and academic performance. A total of 141 U16 soccer players were selected (14.7 ± 0.5 years). Data was collected for academic performance, physical and socio-demographic characteristics, and on two occasions, the dimensions of FS (before of a TR and CM). The results showed that the FS dimensions are higher before of the TR than before of the CM (*p* < 0.05) in all playing positions. In clear goals dimension, forwards showed lower scores than other playing positions, and various dimensions had a positive relationship with academic performance. In conclusion, the FS presented in CM is lower in U16 soccer players compared to that presented in TR. This work has contributed to increasing the knowledge of the fluctuation of the FS that negatively influence the soccer player in pre-competition states and the influence of various factors on this construct.

## Introduction

The degree of attention that players demonstrate during training time (TR) is important for adequate learning and development [1]. This attention is very closely related to what the scientific community calls Flow State (FS), which can be defined as an optimum psychological state. It is a multidimensional concept, a condition where the athlete approaches a situation or motor task under the best psychological conditions, and which implies new characteristics or dimensions (balance between challenge and skills, merging of action and awareness, clear goals, unambiguous feedback, concentration on the task at hand, sense of control, loss of self-consciousness, distortion of the sense of time, autotelic experience) [2, 3]. Subsequently, the dimensions challenge-skills balance, clear goals, and unambiguous feedback were no longer considered as dimensions in themselves, but instead, as pre-conditions for FS [4]. In this vein, Montero [5] describes how some dimensions or characteristics could be considered as precursors of FS while others would be considered as consequences. This author proposes that the three aforementioned dimensions be considered as pre-conditions and that the dimensions themselves be action-awareness merging, concentration on the task at hand, loss of self-consciousness, and autotelic experience, while transformation of time and autotelic experience would be consequences of flow, with autotelic experience falling between being a dimension in and of itself and also a consequence of FS.

FS allows individuals to experience, in a positive way, their own sensations, perceptions and actions in association with certain functions such as cognition, emotion, fixing objectives and rewards processing [6]. Given that athletes and trainers seek to optimize sports performance through multiple factors [7], FS is a relevant object of study for accomplishing this goal [8].

The experience of athletes along with their motivational orientation positively influences focus of attention, and consequently, sports performance [8]. Anxiety and stress are factors which act against this attention and therefore against sports performance [9]. Both experienced players and developing players are susceptible to the influence of these psychological abilities on sports performance, but the latter are even more so [10]. For this reason, the need exists to evaluate psychological abilities and to incorporate this evaluation in TR in order to improve sports performance [11]. These limiting factors lead us to the hypothesis that developing players are more susceptible to competition time (CM) [10], with varying baseline psychological states which might result in lower positive psychological characteristics, such as attention, concentration, self-confidence, and self-esteem.

Players are classified in their playing position according to their physical and physiological characteristics [12], their morphological and anthropometric characteristics, their somatotype [13] and their psychological characteristics, such as impulsiveness [14]. These characteristics impact the possibility of becoming a professional soccer player [15]. Olmedilla et al. [16] conclude that goalkeepers have a better profile and psychological characteristics in comparison to the other playing positions. In indoor-soccer, players in offensive roles are more impulsive than goalkeepers and players in defensive positions [14]. The latter two act with more premeditation [17] which allows them to make individual and collective decisions which avoid unnecessary risks.

In regard to academic performance, as is the case for executive functions, higher academic performance corresponds with more sporting success in developing players [17]. The transition from the junior player to professional should follow an integral and multidimensional development [18], one which includes psychological, academic and sporting aspects. The ongoing practice of physical activity can improve mental and attentional processes, such as dimensions of FS, and consequently academic performance [19, 20]. When participants from the same environment are evaluated and have the same level of physical activity practice, the differences could be verified according to their physical characteristics, which could influence the adolescent's personality [21]. Academic self-concept seems to be positively related to physical characteristics, which, in turn, are the consequences of poor healthy habits [22].

The first aim is to evaluate the variation in FS dimensions between the moment TR begins (baseline) and the moment of official CM as a function of playing position and to determine the FS dimensions profile. The second aim is to explore the relationships between FS dimensions and physical characteristics, and academic performance.

## Materials and methods

### Participants

The subjects of this study were 147 Spanish males in U16 category (14.7 ± 0.50 years, 1.70 ± 0.72 m of height, 61.6 ± 10.0 kg of weight) who participated weekly in official CM. The competitive level of them was medium-high (Second and Third Division over 4 categories). The inclusion criteria were: age between 14 and 16 years, male, no pathology which could alter the results in the psychosocial aspect, and no injuries in the last six months. These last two criteria of not having any pathology and injury were added due to changes and alterations in mood states, anxiety, motivation, among other parameters, by soccer players who have an injury such as an ankle sprain, and pathologies such as a cold or a temporary condition that avoids normal sports practice. These criteria were asked in an ad-hoc questionnaire along with other questions that will be detailed in the instruments section. Previously, one-hundred sixty players were recruited, but thirteen of them did not complete all the questionnaires in all their stages (TR and CM) due to injuries, or not called for the match by technical decision.

### Instruments

With the objective of evaluating FS of the players, the Flow State Scale [23] (translated and validated by García-Calvo et al. [8]) was administered. This questionnaire consists of 36 items concerning thoughts, sensations, and feelings related to the best experience a player has had in a TR and in a CM. Answers are given on a Likert scale from 1 to 10 (1 = completely disagree, 10 = completely agree). The scale includes nine FS dimensions: challenge-skill balance (e.g. “I was challenged, but I believed my skills would allow me to meet the challenge”), action-awareness merging (e.g. “I made the correct movements without thinking about trying to do so”), clear goals (e.g. “I knew clearly what I wanted to do”), unambiguous feedback (e.g. “It was really clear to me how my performance was going”), concentration on the task at hand (e.g. “My attention was focused entirely on what I was doing”), sense of control (e.g. “I had a sense of control over what I was doing”), loss of self-consciousness (e.g. “I was not concerned with what others may have been thinking of me”), transformation of time (e.g. “Time seemed to alter either slowed down or speeded up”) and autotelic experience (e.g. “I found the experience extremely rewarding”). The sum of points for all items gives an overall FS scores [8]. The value total of each dimension was of 40 points, because each dimension was composed of 4 questions from the test. The reliability index was 0.92 (Cronbach's alpha), with all the dimensions greater than 0.70 (except for the transformation of time, with 0.57; Cervelló-Gimeno et al. [24]. In the present study, the reliability on FS dimensions of young soccer player was an average of Cronbach's alpha = 0.80, and all dimensions had an alpha value greater than 0.60.

An ad-hoc questionnaire with questions about socio-demographic variables (age, playing position, team, years federated [experience], hours in TR, previous injuries [inclusion criteria mentioned previously], the presence of family members among the spectators at a match [95% of the total]) was also administered. The independent variable, playing position, was divided into four elements using a modification of the initial classification made by Cárdenas-Fernández et al. [13], i.e. goalkeepers, defenders, midfielders, and forwards.

To record weight and fat mass, the researchers used the TANITA scale (SC-330, Tokyo, Japan). To measure the height, a conventional tape-measure was used, overlapped on the wall.

### Design and procedures

This study was cross-sectional/transverse, descriptive and inferential, and had the duration of a sports season (2016–2017). The same players were evaluated on different occasions although no independent variable was manipulated. All the players were evaluated during the months of October and November, in the middle of the competitive period. The TR and CM were developed according to the planning of the technical staff. Therefore, data was sampled on three separate times. At the first time, the ad-hoc socio-demographic questionnaire and the FS scale (establishing the baseline level) were administered during a TR at least 48 hours before an official CM and the FS scale was again administered during a TR at least 48 hours after an official CM [14]. At the second time, of a pre-competition moment, the FS scale was administered 24 hours before the official CM. On the third time, to evaluate academic performance, report cards from the participants' schools were collected after the first evaluation (January 2017).

Before implementing this study, permission was requested to gather data from the clubs. The participants' parents signed voluntary consent forms. The questionnaires met all the guidelines established by the Helsinki Declaration (2013) on human research. The implementation of this study was approved by the Ethics Committee of the University of Granada (471/CEIH/2018).

### Statistical analysis

The statistical package SPSS for Windows V.22 (IBM SPSS Statistic, Chicago, USA) and Microsoft Office Excel (Microsoft Corp., Redmond, Washington, USA) were used. Firstly, the Kolmogorov-Smirnov test of was performed in order to check the normality of the dependent variables. Then, the t-test for paired samples (in TR and CM times) and the one-way ANOVA test (with the playing position as factor) were carried. In the same time, the Levene test was performed to analyze the homogeneity, and the Bonferroni post-hoc adjustment was used to verify the location of the differences. The threshold values for the size of effect for *t*-test and ANOVA were: small, 0.20 and 0.10; moderate, 0.50 and 0.25; and large, 0.80 and 0.40, respectively [25]. Furthermore, MANOVA test was assessed for comparisons between different playing positions during TR and CM. Lastly, the relationships between the FS variables and academic performance, physical characteristics, and TR hours per week (Pearson *r* and stepwise linear regressions) were analyzed. The level of significance was established at *p* < 0.05.

## Results

**Fig 1:**
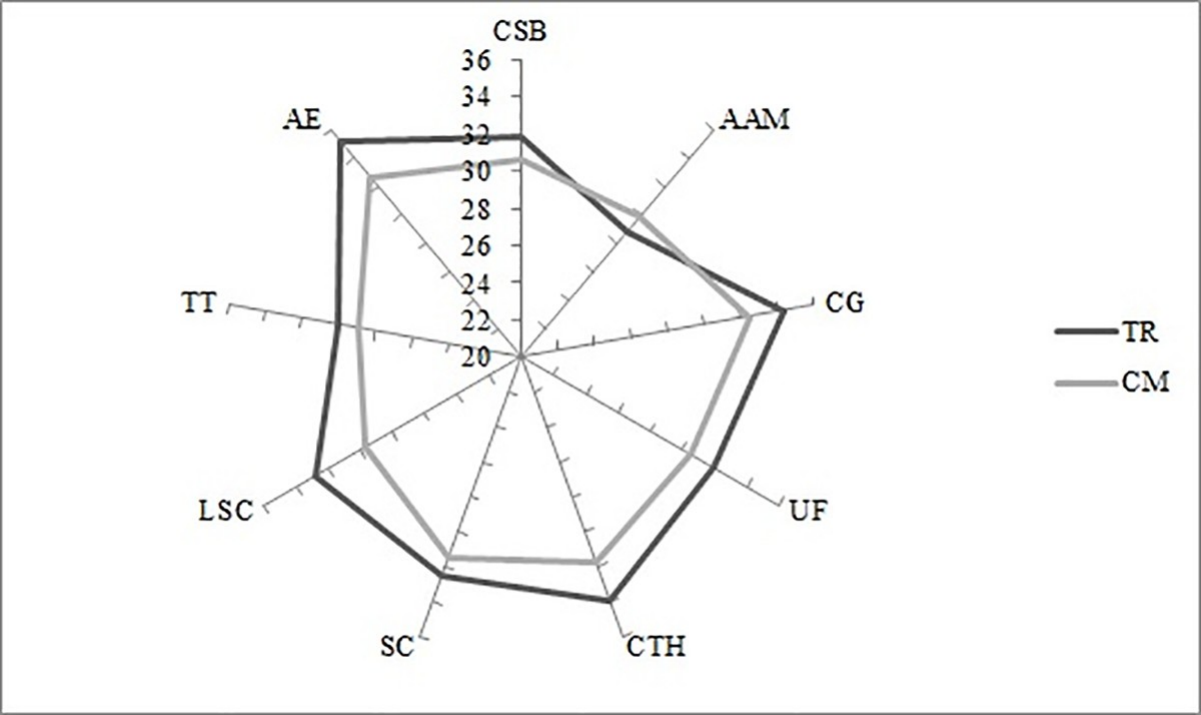
FS profile of U16 soccer players in TR and CM time. CSB: Challenge-skill balance; AAM: Action-awareness merging; CG: Clear goals; UF: Unambiguous feedback; CTH: Concentration on the task at hand; SC: Sense of control; LSC: Loss of self-consciousness; TT: Transformation of time; AE: Autotelic experience.

The results of paired sample tests (*t*-values, significance level, and effect size) for FS dimensions and their correlation with academic performance and with physical activities (Pearson´s *r* and significance level) can be seen in Table 1. Previously, the FS dimensions profile of the soccer players in TR and CM was similar (Fig 1), except in concentration (33.9±4.0 and 31.8±5.4 points; respectively, *p* < 0.05, *d* = 0.40) and autotelic experience (35.1±4.9 and 32.6±5.7 points; respectively, *p* < 0.05; *d* = 0.41). It can be seen that the players have a lower FS profile in official CM in relation to TR. Moreover, there are several inverse correlations in baseline moment (TR) between TR hours per week and the challenge-skill balance, unambiguous feedback, and loss of self-consciousness dimensions. Furthermore, clear goals dimension correlated positively with fat mass and height, and inversely with academic performance in math. Finally, the height was also correlated positively with challenge-skill balance, clear goals, unambiguous feedback, sense of control, loss of self-consciousness, and autotelic experience in CM (*r* = 0.42; 0.35; 0.44; 0.41; 0.37; 0.46; *p* < 0.05; respectively).

**Table 1:** Correlations between academic and physical characteristics and paired-samples T- test of FS dimensions between TR and CM time.

		MAT	PE	EN	Weight	FM	Height	Exp	Training	*T*_*(1*,*132)*_	*p*	*d*
CSB	TR	-0.12**	0.02*	-0.22**	-0.10**	-0.04**	0.22**	-0.02**	-0.32**	1.018	0.321	0.18
	CM	0.28**	0.39*	0.09**	0.16**	-0.01**	0.42**	-0.22**	0.23**			
AAM	TR	-0.28**	-0.29*	0.03**	-0.09**	0.09**	0.13**	-0.36**	-0.26**	0.742	0.396	0.15
	CM	0.07**	0.01*	0.15**	0.09**	0.00**	0.31**	-0.22**	0.14**			
CG	TR	-0.40**	-0.11*	-0.15**	0.18**	0.33**	0.33**	-0.11**	-0.08**	1.693	0.202	0.22
	CM	-0.03**	0.13*	-0.04**	0.20**	-0.04**	0.35**	-0.02**	0.24**			
UF	TR	-0.22**	-0.08*	-0.04**	-0.15**	-0.12**	0.13**	-0.11**	-0.30**	1.512	0.228	0.21
	CM	0.20**	0.30*	0.04**	0.10**	-0.05**	0.44**	0.01**	0.39**			
CTH	TR	-0.07**	0.07*	-0.05**	0.11**	0.06**	0.31**	-0.07**	-0.04**	6.018	0.020	0.40
	CM	0.24**	0.20*	0.07**	0.34**	0.06**	0.31**	-0.12**	0.10**			
SC	TR	-0.27**	-0.13*	-0.03**	-0.03**	0.06**	0.01**	-0.03**	-0.11**	0.714	0.404	0.15
	CM	0.13**	0.12*	0.18**	0.31**	0.13**	0.41**	-0.02**	0.43**			
LSC	TR	-0.13**	-0.04*	-0.21**	-0.23**	-0.19**	0.00**	-0.15**	-0.35**	3.248	0.081	0.31
	CM	0.53**	0.26*	0.40**	0.19**	0.09**	0.37**	0.09**	0.28**			
TT	TR	0.20**	0.06*	0.14**	-0.04**	-0.12**	0.17**	-0.02**	-0.15**	0.522	0.475	0.13
	CM	0.31**	0.30*	0.24**	0.05**	-0.13**	0.14**	-0.13**	0.05**			
AE	TR	0.08**	0.19*	0.03**	0.01**	-0.07**	0.18**	-0.14**	-0.04**	6.519	0.016	0.41
	CM	0.29**	0.34*	0.27**	0.26**	0.01**	0.46**	-0.10**	0.28**			

* Correlation *p* < 0.05

** Correlation *p* < 0.01; TR: Training time; CM: Competitive time; CSB: Challenge-skill balance; AAM: Action-awareness merging; CG: Clear goals; UF: Unambiguous feedback; CTH: Concentration on the task at hand; SC: Sense of control; LSC: Loss of self-consciousness; TT: Transformation of time; AE: Autotelic experience; MAT: Academic performance in math; PE: Academic performance in Physical Education; EN: Academic performance in English language; FM: Fat mass; Exp: Experience; Training: Week training.

Table 2 shows the results of FS dimensions as a function of playing position in baseline TR and in CM. It can be seen that in TR, goalkeepers have higher values in clear goals and unambiguous feedback; defenders, in challenge-skill balance and concentration; and midfielders, in clear goals, sense of control, and autotelic experience. In ANOVA, midfielders had higher values for clear goals (*p* < 0.05).

**Table 2:** One-way ANOVA test of FS dimensions between TR and CM according to playing position and *t*-test between TR and CM.

		Goalkeepers *(N* = 27)			Defenders (*N* = 38)			Midfielder (*N* = 44)			Forwards (*N* = 36)			*F*_(3.142)_	*p*	*η*^*2*^_*p*_
CSB	TR	30.50	±	4.51	32.64	±	5.97*	33.19	±	5.62	31.17	±	4.91	0.472	0.704	0.11
	CM	31.75	±	4.11	27.33	±	5.07*	32.00	±	4.00	31.50	±	4.34	2.295	0.098	0.25
AAM	TR	29.75	±	5.56	29.00	±	6.53	29.88	±	6.71	29.42	±	4.42	0.049	0.985	0.03
	CM	32.75	±	4.92	28.33	±	7.11	29.38	±	6.73	30.75	±	5.29	0.523	0.669	0.12
CG	TR	36.25	±	3.30*	32.45	±	8.49	37.06	±	2.79*^FO^	32.25	±	3.65^**MI**^	2.865	0.049	0.25
	CM	31.75	±	4.65*	31.33	±	6.19	33.15	±	5.34*	32.13	±	4.09	0.233	0.873	0.08
UF	TR	35.00	±	3.46*	32.36	±	5.52	32.06	±	5.89	31.50	±	5.44	1.506	0.228	0.19
	CM	29.00	±	3.37*	29.11	±	6.74	31.62	±	5.16	31.25	±	2.38	0.621	0.607	0.13
CTH	TR	33.00	±	3.74	34.64	±	3.41*	35.06	±	3.44	32.50	±	4.83	1.172	0.333	0.16
	CM	31.75	±	6.60	30.33	±	6.48*	32.62	±	4.43	32.50	±	5.32	0.352	0.788	0.10
SC	TR	35.25	±	3.30	32.55	±	5.92	33.63	±	4.13*	30.00	±	6.35	0.413	0.745	0.10
	CM	33.25	±	7.04	30.33	±	5.68	31.00	±	6.34*	33.38	±	3.70	0.560	0.646	0.13
LSC	TR	32.00	±	2.94	34.82	±	5.12	33.13	±	6.54	31.58	±	7.19	0.561	0.644	0.11
	CM	27.50	±	8.27	30.67	±	7.21	29.23	±	7.27	31.50	±	5.81	0.366	0.778	0.10
TT	TR	30.75	±	4.19	29.18	±	7.53	30.31	±	7.49	30.75	±	6.08	0.114	0.951	0.05
	CM	28.00	±	4.40	28.33	±	8.35	28.31	±	9.61	31.63	±	5.21	0.362	0.781	0.10
AE	TR	34.00	±	3.74	34.73	±	4.43	36.50	±	4.00*	33.83	±	5.62	0.896	0.452	0.14
	CM	31.50	±	8.54	30.67	±	5.90	33.31	±	5.31*	35.13	±	4.55	0.962	0.424	0.17

* *p* < 0.05 between TR and CM time for each playing position; TR: Training time; CM: Competitive time; CSB: Challenge-skill balance; AAM: Action-awareness merging; CG: Clear goals; UF: Unambiguous feedback; CTH: Concentration on the task at hand; SC: Sense of control; LSC: Loss of self-consciousness; TT: Transformation of time; AE: Autotelic experience.

MANOVA test assessed the FS dimensions with the playing positions (goalkeepers, defenders, midfielders, and forwards) and the time (TR and CM) as factors. There was no interaction between the indicated factors (*p* < 0.05). Lastly, various linear relationships were established between FS dimensions and physical characteristics and academic performance. It is notable that clear goals is predicted by height and mathematics performance with 29% of the explained variance (*p* = 0.004; SEE = 5.103; Table 3), and challenge-skill balance dimension is predicted by training week (in hours) with 20% of the explained variance (*p* = 0.012; SEE = 5.074). The dimensions of action-awareness merging, concentration on the task at hand, transformation of time, and autotelic experience did not offer any significant model.

**Table 3:** Linear regression test (stepwise) of FS, physical characteristics, and academic performance.

DV	IV	Time	*R*^*2*^	*SEE*	*F*_*(1*,*141)*_	*p*	EQUATION
LSC	Training	TR	0.14	5.717	6.823	0.013	LSC = 42.041 + (Training x -2.009)
UF	Training	CM	0.13	4.661	4.929	0.034	UF = 22.816 + (Training x 1.645)
UF	Training	TR	0.14	5.329	4.427	0.044	UF = 45.469 + (Training x -2.879)
SC	Training	TR	0.17	5.285	5.902	0.022	SC = 48.185 + (Training x -3.297)
SC	Training	CM	0.14	4.997	4.432	0.044	SC = 17.838 + (Training x 2.992)
CSB	Training	TR	0.20	5.074	7.148	0.012	CSB = 48.223 + (Training x -3.484)
CG	HE-MAT	TR	0.29	5.103	6.951	0.004	CG = -34.054 + (HE x 0.429) + (MAT x -0.922)
LSC	MAT	CM	0.26	6.125	10.039	0.004	LSC = 22.234 + (MAT x 1.531)

DV: Dependent variable; IV: independent variable; CSB: Challenge-skill balance; CG: Clear goals; UF: Unambiguous feedback; SC: Sense of control; LSC: Loss of self-consciousness; TR: Training time; CM: Competitive time; HE: Height; Training: Week training; MAT: Academic performance in math; SEE: Standard Error of Estimation.

## Discussion

The objectives of this study were, first, to evaluate the variations in FS dimensions in young soccer players between the moment of TR and the moment of official CM, as a function of their playing position, and in parallel, determine their FS dimensions profile; second, to find relationships between the FS dimensions and the players' physical characteristics, between FS dimensions and academic performance. The results show that U16 soccer players have lower FS, in general, in the moment of official CM as compared to the scores found during TR. This could be due to the fact that players beginning a sports career perceive CM as a source of stress and anxiety, expressed in cognitive anxiety, consequently causing a decreased motivation and concentration [26], produced, in part, by fear of failure [27], and in part, owing to the exposure to and pressure from the public in CM matches and the presence of family members among the spectators [28]. It is an environment where the young players are socially evaluated [29] and the pressure they feel comes from their anticipation of success and their desire for an immediate win. In addition, it has been verified that soccer players do not train as they compete [30], that is, that physically and physiologically, the players have higher responses in CM. This fact could bring out a degree of fatigue far superior to that of TR, which would cause discouragement or apathy moments before sports CM.

Accordingly, the soccer player's FS profile shows fluctuations between the two contexts, with higher FS scores in TR sessions. This may be attributed to various factors: the developing players associating these sessions with moments of playfulness and lack of inhibition among the team members [31]; TRs organized in a more constructive way which avoid a traditional methodology, namely, mechanical, technical and repetitive [32]; and the way the coach interacts with the players, the player's experience in TR, and the context of learning [33].

On the other hand, the players showed similar scores in FS dimensions across the different playing positions, with the exception of the forwards who showed lower scores in clear goals (*P* < .05). This could be due to the fact that offensive players have a distinctive personality, demonstrating significantly higher levels of impulsivity in futsal players [14], less social, more aggressive and with less behavior control in soccer players [34] than defensive players, whose position demands more concentration [35], fundamentally aimed at recovering the ball [36]. All the playing positions showed higher FS scores in TR than in CM. Thus, the initial hypothesis is verified. Developing soccer players are psychologically more susceptible in CM, and consequently experience a reduction in positive psychological characteristics, in this case, FS.

The clear goals, balance between challenge and skills, and loss of self-consciousness dimensions are positively associated with the academic performance of the players. This is to be expected as various dimensions of FS are negatively associated with procrastination, that is, the tendency to constantly delay the execution of a task [37]. Positive relationships were found between the dimensions challenge-skills balance, direct and immediate feedback, and sense of control in TR time. In this light, a greater number of TR sessions could increase the player's self-concept [38], with a subsequent gain in attention. On another note, height and academic performance in mathematics predicted, with a 29% of explained variance, the dimension clear goals. A player with higher grades in mathematics will have better logical-mathematical reasoning [39], which may contribute to his obtaining higher results in the dimension clear goals and to his facility to resolve situations in small-sided games, both in TR and in CM [40].

A possible limitation of this study, which at the same time could serve as a suggestion for future lines of research, was not collecting information relative to the motivational orientation of the players, or their perception of success. Having this information would provide a fuller psychological profile of the developing soccer player, which the coach could use for the player's benefit. Furthermore, these results present the problem of being dependent on the adolescent's interoceptive capacity, on the one hand, and of being subject to possible social desirability bias, on the other hand. Beyond these self-reported data, we do not have information for scientific knowledge from research that contrasts the ability to control the FS perceived by the adolescent with their academic performance and physical characteristics measured moments before the CM and TR. Regarding the use of the baseline sample 48 hours after the CM and 48 hours before, the indications of previous studies of self-reported psychological response have been followed [14, 41].

As practical applications, the results of this study could be considered of special interest to the professionals of the sport of soccer (coach, physical trainer, sports psychologist, among others) since a decrease in the attention variables or FS dimensions is perceived in soccer players in developing age due to sports CM, which could be carried out psychological intervention programs in TR to reduce the effect of CM on young soccer players [25]. Regarding academic performance, it is necessary to approach a comprehensive education, taking into account the psychological fluctuations that occur with the educational transition [42]. In this way, there should be coordination between the elementary-school, middle-school, high-school and university, avoiding major changes for students.

## Conclusions

In conclusion, the main finding of this study was that U16 soccer players showed lower FS values during CM in all the dimensions, so it is necessary to add psychological training tasks in TR sessions in order to prepare the soccer players comprehensively, while avoiding the effect of CM. It was also found that forwards had lower values in clear goals in comparison with the other playing positions. Finally, it was found that in TR, academic performance and physical characteristics, both have a direct relationship with FS dimensions, suggesting that family members and coaches encourage the player to maintain good academic performance in the interest of sports performance.

## Supporting information

S1 Data (SAV)
